# Olfaction and Environment: Tsimane’ of Bolivian Rainforest Have Lower Threshold of Odor Detection Than Industrialized German People

**DOI:** 10.1371/journal.pone.0069203

**Published:** 2013-07-29

**Authors:** Agnieszka Sorokowska, Piotr Sorokowski, Thomas Hummel, Tomas Huanca

**Affiliations:** 1 Institute of psychology, University of Wroclaw, Wrocław, Poland; 2 Smell & Taste Clinic, Department of Otorhinolaryngology, Technical University of Dresden, Dresden, Germany; 3 Centro boliviano de Investigación y de Desarrollo Socio Integral, Correo Central, San Borja, Beni, Bolivia; University of Arizona, United States of America

## Abstract

Olfactory sensitivity varies between individuals. However, data regarding cross-cultural and inter-group differences are scarce. We compared the thresholds of odor detection of the traditional society of Tsimane’ (native Amazonians of the Bolivian rainforest; *n* = 151) and people living in Dresden (Germany; *n* = 286) using “Sniffin’ Sticks” threshold subtest. Tsimane’ detected *n*-butanol at significantly lower concentrations than the German subjects. The distribution of thresholds of the Tsimane’ was very specific, with 25% of Tsimane’ obtaining better results in the olfactory test than any member of the German group. These data suggest that differences in olfactory sensitivity seem to be especially salient between industrialized and non-industrialized populations inhabiting different environmental conditions. We hypothesize that the possible sources of such differences are: (i) the impact of pollution which impairs the olfactory abilities of people from industrialized countries; (ii) better training of olfaction because of the higher importance of smell in traditional populations; (iii) environmental pressures shaping olfactory abilities in these populations.

## Introduction

For humans, olfaction contributes significantly to perception of the environment. It enables people to learn the odors relevant to particular life experiences and their specific environment [1]. Some authors have categorized the use of smell in three main groups [2]: related to ingestive behavior [3,4], avoiding environmental hazards [5], and social communication [6,7]. Also, a decrease in the quality of life resulting from olfactory dysfunction hints at the importance of smell [8].

Olfactory sensitivity varies between individuals. Subjects’ sensitivity to odors can differ dramatically [9] as do assessments of the perceived quality and pleasantness of certain odors [10]. Inter-individual differences can be general [9] or they can concern specific odors [11]. Differences in general olfactory acuity can result from many factors, such as age [12,13], sex, smoking habits, or body weight of an individual [12]. Olfactory dysfunctions, such as those resulting from environmental pollution [14], neurodegenerative diseases [15] or infections [16] might also account for differences in olfactory acuity.

During earlier periods of human evolution, olfaction might have been more important than it is today. Many authors suggest that the capability of the chemosensory system involved in olfactory perception has declined in the primate lineage in comparison with other mammals [17]. Gilad and colleagues [18] suggested that the olfactory *repertoire* deteriorated with the evolution of full trichromatic vision. Thus, olfaction in humans seems to be of less importance than it is for other mammals [2]. Likewise, the high prevalence of smell dysfunctions in humans might suggest the decreasing importance of the olfactory system [19], although this could be due largely to the increase in the average age of modern human populations.

Drawing on all the aforementioned findings we suggest that, in addition to inter-individual diversity, differences in olfactory abilities should exist across different populations. However, studies focusing on this matter are very scarce and, to our knowledge, none have analyzed the olfaction of populations living in natural environmental conditions. We hypothesize that the olfactory abilities of indigenous people who live in such conditions should be better compared with people in Western societies. We suggest three possible mechanisms which could cause such differences. First, in populations living in natural environmental conditions, the sense of smell might be better trained because smell is more important in situations related to, for example, ingestive behavior or avoiding environmental hazards. Second, non-industrialized societies are not subject to industrial pollution, which presumably impairs the sense of smell of people from industrialized countries. Finally, environmental pressures shaping olfactory abilities in indigenous populations could have improved their sense of smell beyond the abilities of modern, industrialized groups.

To test our hypothesis, we compared the thresholds of odor detection in two populations: Germans living in Dresden and the traditional society of Tsimane’ (Bolivian Amazon). The latter are forager-horticulturists whose subsistence centers on hunting, fishing, and slash-and-burn agriculture. The Tsimane’ number ~8,000 people and live in ~120 villages along the Maniqui River, Department of Beni, Bolivia.

## Materials and Methods

### Ethical approval of the study protocol

The study was conducted according to the principles expressed in the Declaration of Helsinki. The study protocol and consent procedure received ethical approval from the Institutional Review Board (IRB) of the University of Wroclaw (Wrocław, Poland) and from the Great Tsimane’ Council (the governing body of the Tsimane’). Participants provided informed consent before study inclusion. Due to the low levels of literacy of the Tsimane’, we obtained oral consent for participation and documented it using a portable recorder.

### Participants

We conducted the first phase of the study among 151 Tsimane’: 77 females aged 18-53 years (*M*=28.53, *SD*=9.16) and 74 men aged 18-50 years (*M*=32.59 years, *SD*=11.75) from six villages along the Maniqui River. None of them reported otoloryngological problems at the time of the study. They received a gift (household items worth ~six USD) for participating in a series of studies. In the second part of the study we used data from 286 non-smoking people from Dresden (published previously [20]). We took data from all German subjects who matched in terms of age, sex and smoking status to the Tsimane’: –166 females aged 18-49 years (*M*=25.75, *SD*=7.49) and 120 males aged 18-50 years (*M*=31.15, *SD*=11.44). We included only non-smoking individuals from Germany because smoking is related to an increased risk of smell impairment [21] and because the Tsimane’ do not smoke. Data used in both parts of the study are available upon request from the corresponding author.

### Procedure

We assessed the olfaction of participants using a “Sniffin’ Sticks” threshold subtest (Burghart Messtechnik, Wedel, Germany) [22]. Differences in olfactory performance can result from cultural factors, such as the physical availability of given odors [23]. Consequently, olfactory abilities with regard to the recognition of certain smells can differ between groups [24] or cultures [25]. The discrimination and identification subtests contain smells which might be known only in industrialized populations, so we did not use them. The odorant used in the threshold test (*n*-butanol) was not common for both study groups.

We assessed odor thresholds using a single-staircase, three alternative forced choice (3-AFC) procedure (for details see [22]:). The scores in the test can range between 1 and 16 and represent a geometric series of concentrations starting from a 4% *n-*butanol solution in pen number 1 to 2.7877 × 10^-9^ in pen number 16 (dilution ratio 1:2 in deionized aqua conservata as a solvent). The threshold was the mean of the last four of seven staircase reversals. Trained experimenters conducted the test on an individual basis. A translator explained the procedure to the participants in their native languages. We ensured that the Tsimane’ participants understood the procedure before completing the task. That is, we asked them to smell the pen number 1 containing the odor (the 4% concentration of *n*-butanol), and when they confirmed that they had smelled it, we asked them to choose this smelling pen from the set of three pens. We excluded a few people who did not understand the task from further participation in the study.

## Results

Thresholds in the Tsimane’ group were not normally distributed (Shapiro–Wilk W = .917, *p* < .001), we graphed performance data for the two groups as medians and interquartile ranges and compared them using the non-parametric Mann–Whitney *U* test. We undertook two-tailed tests throughout, using STATISTICA ver10 (StatSoft, Inc.) with *p* < .05 as the level of significance.

Descriptive statistics are shown in Table 1 (raw data are available upon request from the corresponding author). Median values in the Tsimane’ and German groups were 10.75 and 8.5, respectively. Subjects from the Tsimane’ group detected *n*-butanol at significantly lower concentrations than did German subjects (Mann–Whitney *U* test: *U* = 16760.5_286,151_, *p* < 0.001, *r* = .184). Based on the median scores, this represented a two- to four-fold difference in the concentration needed to detect the stimulus. The scores for males and females did not differ significantly in the German (*U* = 20076.5_228,189 ,_ *p* = .23) or Tsimane’ groups (*U* = 2494.5_77,74_, *p* = .19).

**Table 1:** Descriptive statistics of odor threshold values in Tsimane’ and German participants.

		Tsimane’ participants	German participants
*N*		151	286
Mean		10.12	8.73
SD		4.69	2.18
Minimum		1	2.5
Maximum		16	14.5
Percentiles	5	1.75	5.06
	10	2.5	6
	25	6.13	7.25
	50	10.75	8.5
	75	14.63	9.94
	90	15.75	11.75
	95	16	12.5

**Figure 1:**
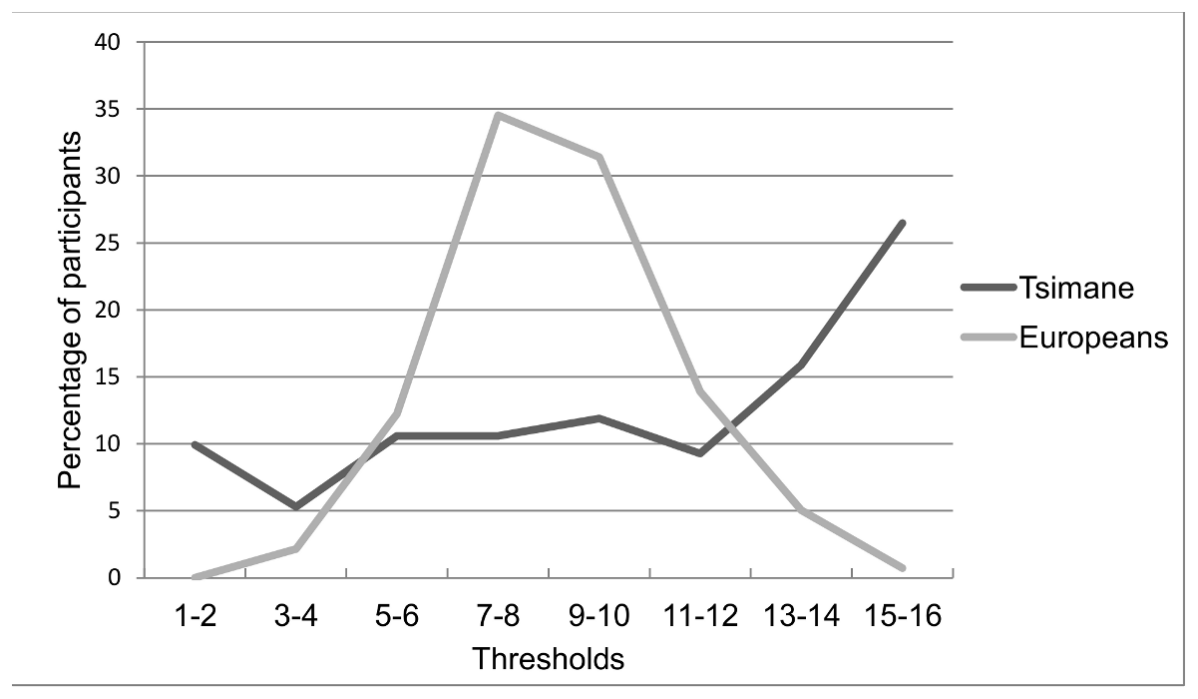
Distributions of scores in odor threshold test for Tsimane’ and European participants. The scores in the test can range between 1 and 16 and they represent geometric series of concentrations of *n-*butanol (ranging from a 0.04 to 2.7877 × 10^–9^ *n-*butanol solution, dilution ratio 1:2).

**Figure 2:**
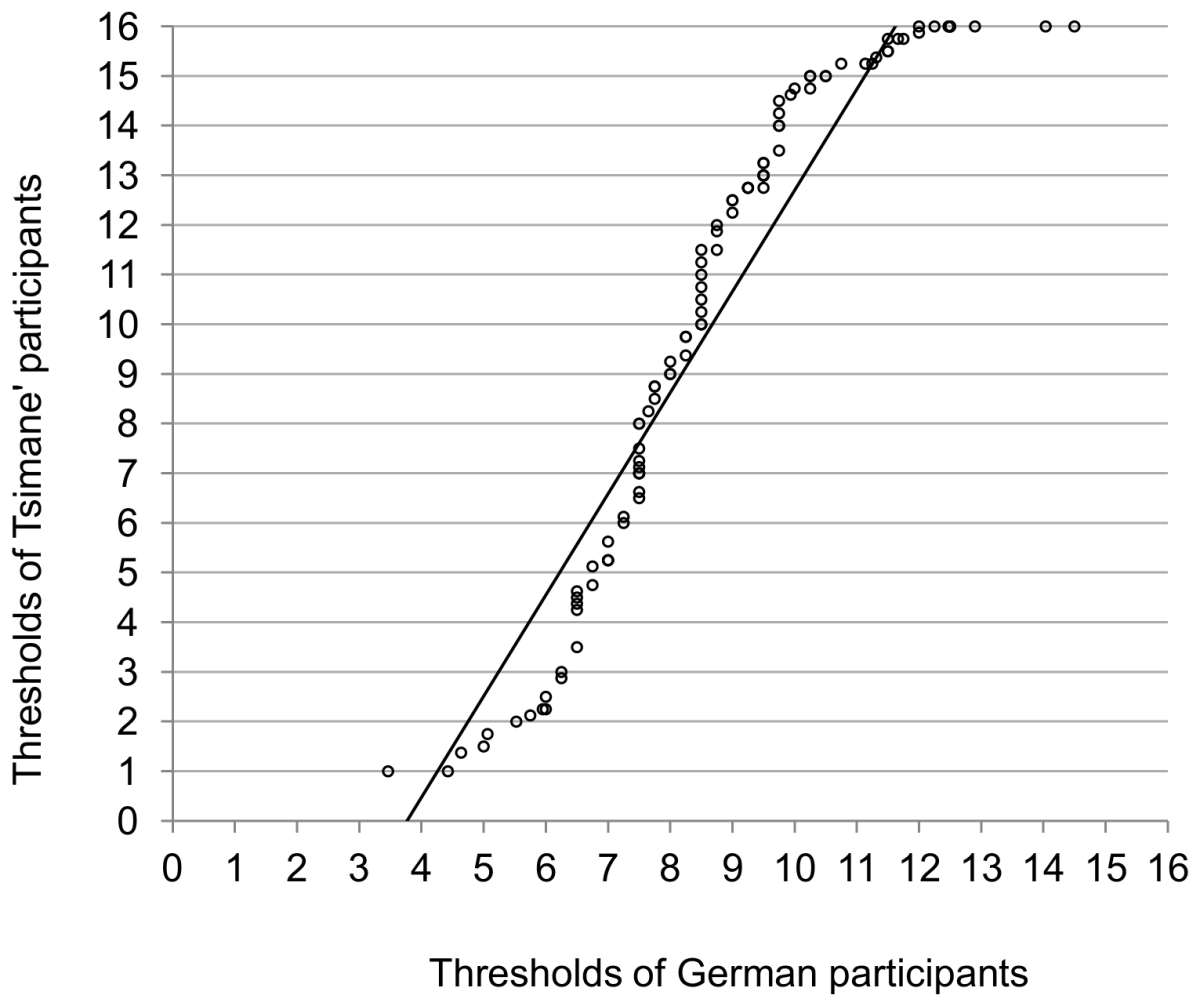
Quantile-Quantile (Q-Q) plot of odor thresholds for Tsimane’ and German participants. The solid line represents the general trend of the Q–Q plot.

Figure 1 shows the distributions of odor thresholds for Tsimane’ and German participants. We compared them graphically using quantile-quantile (Q-Q) plot [26] (Figure 2). The Q–Q plot demonstrated that the distributions in the two groups differed. The distribution of results from the Tsimane’ participants’ was more dispersed than the distribution of results from the German participants’. Also, the Q–Q plot demonstrated an S-shaped curve with outliers apparent in the upper tail. This finding suggested that the distribution of results from the Tsimane’ population was more skewed and that it had heavier tails than the results from the German group.

## Discussion

We found that the Tsimane’ people, living in natural environmental conditions, had significantly lower thresholds of odor detection than those of subjects from an industrialized population of Europeans. In addition to being low, the thresholds of the Tsimane’ population were more widely distributed. That is, 25% of Tsimane’ obtained higher results, and 10% of Tsimane’ obtained lower results in the olfactory test than did any member of the German group. Our results suggest also that some Tsimane’ might have even lower thresholds than those tested the method described here.

The low average thresholds observed among the Tsimane’ are even more striking if we consider the background of the two groups participating in the study. The German group comprised healthy, non-smoking adults whose socioeconomic background ensured professional health care starting in the moment of their conception and lasting throughout their lives. None of the villages where we conducted the study had a permanent physician or a nurse (people had to travel between a few hours and a few days by boat to reach the closest physician), the participants did not have access to modern medicines, and a large part of their society suffered from diseases of the upper respiratory tract every time the weather got colder. In general, despite their relatively good nutrition, illnesses are very common among the Tsimane’. The TAPS Group noted that 45% of adults reported being ill within the previous 7 days and between the years of 2003 and 2007, and that adults reported that illness had restricted their activity for an average of 1.2–2.2 days in a 2-week recall period, depending on the year [TAPS database (2002-7) Retrieved 18 February 2013 from www.Tsimane’.org]. Also medical examinations in 2004 and 2005 showed that >60% of Tsimane’ had symptoms of a gastrointestinal or respiratory illness [27].

In our study, 24% of Tsimane’ subjects obtained scores lower or equal to 6 (representing a 0.016461% dilution of *n*-butanol), whereas such a result was obtained by in only 10% of the German cohort, and 10% of Tsimane’ scored lower than did any member of the German group. Upper respiratory tract infections (UTRIs)-typically associated with the common cold or influenza, and chronic rhinosinusitis (CRS)-enduring inflammation in the mucosa of the nose and paranasal sinuses, are frequent causes of olfactory loss [28–30]. Such olfactory disorders tend to be transient, but certain symptoms may be irreversible resulting in permanent *Parosmia* , phantosmia, hyposmia, or anosmia [29]. Studies report that between 11% and 40% of all cases of smell loss are caused by URTIs and CRS[16]. . Also, Reden and collaborators [31] showed that the smell of only 47% of patients aged <40 years improved during ~1 year of observation after an URTI, and this percentage was even lower (7%) for patients aged ≥70 years. The Tsimane’ reported being healthy at the time of the study but, in the past, they might have suffered from otorhinolaryngological or other diseases impairing olfaction [19]. We hypothesize that the average score of the Tsimane’ might be even higher if their health and access to medical services were comparable with that of the Germans.

We showed that the olfactory abilities of the Tsimane’ population were exceptionally developed even under the aforementioned conditions. Almost 30% of the participants scored ≥14 points out of 16 (in the German group it was 2%). For some of the Tsimane’ even the most difficult level of the test (level 16: concentration 2.7877 × 10^-9^) appeared extremely easy (personal observation by A.S. and P.S.).

We propose a few explanations of our results: (i) the impact of pollution which impairs the olfactory abilities of people from industrialized countries; (ii) better training of olfaction because of the higher importance of smell in traditional populations; (iii) environmental pressures shaping olfactory abilities in these populations.

### Environmental influences

The first explanation is the influence of environmental factors on the sense of smell of our participants (or rather the non-influence of industrial air pollution on Tsimane’ participants). Studies have shown that pollution has a significant, negative impact on olfactory acuity [14] and pollution is a serious problem in industrialized regions [32]. The German participants clearly lived in much more industrialized surroundings than the Tsimane’, and this could explain (at least in part) the observed differences in olfactory performance. However, in high-income, developed cities (especially in Europe), the concentrations of pollutants is not extremely high, owing to the increasingly strong restrictions which local governments and international organizations impose [32]. Also, the Tsimane’ engage in slash-and-burn agriculture and use wood and leaves to cook food [33]. These actions also generate many pollutants which affect the respiratory tract and olfaction, and potentially expose them to even higher levels of pollutants than would be found in industrialized Germany. Therefore, pollution alone does not provide a full explanation of the differences in detection thresholds we observed. The agricultural and/or cooking practices could lower the potential performance of many Tsimane’ and account for the lowest performance of some participants together with URTIs.

### Training

The second explanation of our results is that the Tsimane’ learned how to smell “more efficiently” because, in the natural environmental conditions in which they live, olfaction is very useful. As Stevenson [2] suggested, in modern societies, olfaction is rarely used for the detection of food. In contrast, olfaction is more useful for the Tsimane’, whose traditional subsistence patterns center on hunting, fishing, gathering, and horticulture. Byron [34] reported that ~71% of foods consumed within a typical Tsimane’ household came from hunting, fishing, and crop products and that 75% of meat consumed in such households was fish or wild game. Additionally, people in modern societies do not need to use olfactory cues to recognize items such as plants, whereas discriminating items by smell is practiced widely in traditional societies. For example, odors are important to the Matsigenka and Yora of the Peruvian Amazon [35] and the Kenyah Leppo` Ke of Borneo [36] for the identification and evaluation of medicinal plants, and Jernigan [37] reported that the Aguaruna Jívaro of the northern Peruvian Amazon use odors to recognize and judge the relatedness of trees found in their local environment. Those studies suggest that, unlike modern societies, in traditional societies olfaction is important in terms of finding and identifying food and medicinal plants. Thus, because smell can improve with training [4] olfaction in such societies (including the Tsimane’) could be more efficient than that in industrialized groups.

### Genetics

Third, general olfactory acuity can have a genetic component [38] and might differ in people of different races [12]. Menashe, Man, Lancet and Gilad [39] showed that non-African individuals had significantly fewer functional olfactory receptors than did African American individuals. This result, as well as the cross-cultural differences in the perception of odors [23], suggests that different evolutionary pressures may have shaped the chemosensory *repertoire* in different human populations. Hypothetically, the decrease in olfactory abilities of the Tsimane’ (and maybe other traditional societies) could have been slower (or less pronounced) than that in industrialized, modern societies.

At this stage of research we cannot determine the exact sources of the high olfactory performance of the Tsimane’. However, our study shows that olfaction might differ between populations and that factors influencing olfactory acuity probably include environmental pressures.
